# (2*E*)-1-(1,3-Benzodioxol-5-yl)-3-(2-bromo­phen­yl)prop-2-en-1-one

**DOI:** 10.1107/S1600536810015552

**Published:** 2010-05-08

**Authors:** Hongqi Li, R. S. Rathore, K. Prakash Kamath, H. S. Yathirajan, B. Narayana

**Affiliations:** a Key Laboratory of Science & Technology of Eco-Textiles, Ministry of Education, College of Chemistry, Chemical Engineering & Biotechnology, Donghua University, Shanghai 201620, People’s Republic of China; b Bioinformatics Infrastructure Facility, School of Life Science, University of Hyderabad, Hyderabad 500 046, India; c Department of Physics, Mangalore University, Mangalagangotri 574 199, India; d Department of Studies in Chemistry, University of Mysore, Manasagangotri, Mysore 570 006, India; e Department of Studies in Chemistry, Mangalore University, Mangalagangotri 574 199, India

## Abstract

The mol­ecule of the title compound, C_16_H_11_BrO_3_, is essentially planar with a maximum deviation of 0.178 (4) Å and the configuration of the keto group with respect to the olefinic double bond is typically *s-cis*. In the crystal structure, inter­molecular Br⋯O inter­actions [3.187 (3)Å] give rise to chains parallel to the *b* axis. Adjacent chains are further linked along the *a* axis by C—H⋯π inter­actions. The crystal studied was a racemic twin with a 0.595 (13):0.405 (13) ratio.

## Related literature

For chalcones, see: Di Carlo *et al.* (1999 ▶); Sarojini *et al.* (2006 ▶); Yarishkin *et al.* (2008 ▶). For halogen-bonding inter­actions, see: Thallapally *et al.* (2002 ▶); Metrangolo *et al.* (2005 ▶); Riley *et al.* (2009 ▶). For related structures, see: Harrison *et al.* (2006 ▶); Rathore *et al.* (2006 ▶); Li *et al.* (2008 ▶); Jasinski *et al.* (2010 ▶). For racemic twinning, see: Flack (1983 ▶); Flack & Bernardinelli (2000 ▶); Gömez *et al.* (2010 ▶).
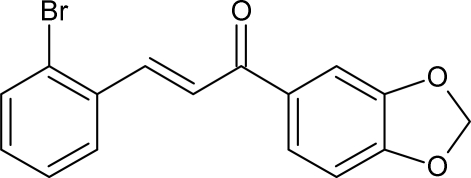


## Experimental

### 

#### Crystal data

C_16_H_11_BrO_3_
*M* *_r_* = 331.16
Orthorhombic, 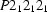

*a* = 5.0434 (2) Å
*b* = 12.9354 (4) Å
*c* = 20.8916 (7) Å
*V* = 1362.93 (8) Å^3^
*Z* = 4
Mo *K*α radiation
μ = 3.02 mm^−1^
*T* = 296 K
0.53 × 0.19 × 0.16 mm

#### Data collection

Bruker SMART APEXII CCD area-detector diffractometer
Absorption correction: multi-scan (*SADABS*; Bruker, 2004 ▶) *T*_min_ = 0.576, *T*_max_ = 0.653
17031 measured reflections
2674 independent reflections
2437 reflections with *I* > 2σ(*I*)
*R*_int_ = 0.023

#### Refinement

*R*[*F* ^2^ > 2σ(*F* ^2^)] = 0.034
*wR*(*F* ^2^) = 0.086
*S* = 1.06
2674 reflections
182 parameters
H-atom parameters constrained
Δρ_max_ = 0.64 e Å^−3^
Δρ_min_ = −0.52 e Å^−3^
Absolute structure: Flack (1983 ▶), 1086 Bijvoet pairs
Flack parameter: 0.595 (13)

### 

Data collection: *APEX2* (Bruker, 2004 ▶); cell refinement: *SAINT-Plus* (Bruker, 2004 ▶); data reduction: *SAINT-Plus*; program(s) used to solve structure: *SHELXS97* (Sheldrick, 2008 ▶); program(s) used to refine structure: *SHELXL97* (Sheldrick, 2008 ▶); molecular graphics: *ORTEP-3* (Farrugia, 1997 ▶); software used to prepare material for publication: *SHELXL97*.

## Supplementary Material

Crystal structure: contains datablocks global, I. DOI: 10.1107/S1600536810015552/rz2432sup1.cif

Structure factors: contains datablocks I. DOI: 10.1107/S1600536810015552/rz2432Isup2.hkl

Additional supplementary materials:  crystallographic information; 3D view; checkCIF report

## Figures and Tables

**Table 1:** Hydrogen-bond geometry (Å, °) *Cg*3 is the centroid of the C10–C15 ring.

*D*—H⋯*A*	*D*—H	H⋯*A*	*D*⋯*A*	*D*—H⋯*A*
C16—H16*B*⋯*Cg*3^i^	0.97	2.76	3.563 (4)	141

Symmetry code: (i) 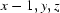.

**Fig. 1.**
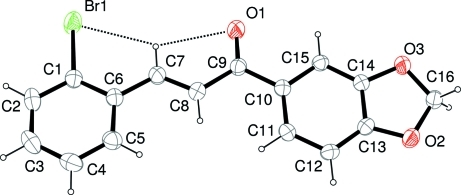
The molecular structure of the title compound. Displacement ellipsoids are drawn at 30% probability level. Dotted lines indicate intramolecular hydrogen bonds.

**Fig. 2.**
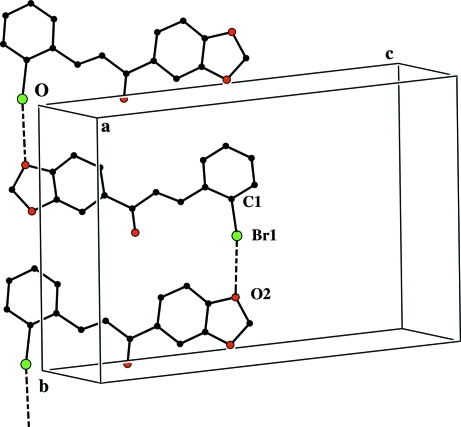
Molecular packing of the title compound showing the halogen···oxygen interactions (dashed lines) forming one-dimensional chains along the b axis.

## supplementary crystallographic information

### Comment

Chalcones possess many interesting biological and pharmacological properties. They are highly reactive substances of varied nature. Recently, it is shown that few of the derivatives are able to block voltage-dependent potassium channels (Yarishkin *et al.*, 2008). Chalcones have been also implicated in organic nonlinear optical materials for their SHG conversion efficiency (Sarojini *et al.*, 2006). The radical quenching property of the phenolic groups present in many chalcones or chalcone-rich plant extracts has led to their use as drugs or food preservatives (Di Carlo *et al.*, 1999). We earlier reported structures of chalcone derivatives (Harrison *et al.*, 2006; Rathore *et al.*, 2006; Jasinski *et al.*, 2010; Li *et al.*, 2008). In continuation of the study, we have synthesized a new chalcone analog, C_16_H_11_BrO_3_, (I), and discuss its crytal structure herein.

The crystal of (I) studied was racemically twinned in a 0.595 (13):0.405 (13) ratio. Similar racemic twinning in Br-containing compounds was observed by Gömez *et al.* (2010). The skeleton of (I) is essentially planar possessing two intramolecular short contacts. The bifurcated C1—H7···(Br1, O1) promote planarity of the molecular skeleton (Table 1). The configuration of the keto group with respect to the olefinic double bond is typically s-cis, with the C7—C8—C9—O1 torsion angle of -1.6 (7)° (Rathore *et al.*, 2006).

Crystal packing is characterized by halogen···oxygen interactions between molecules related by 2-fold screw axis, with the Br1 ···O2^i^ distance of 3.187 (3)Å [symmetry code (i): 0.5+x, 0.5-y, -z] and the C1—Br1···O2 angle of 170.6 (1)°. The Br···O interaction leads to a one-dimensional chain along *b* axis. Crystal packing is shown in Fig 2. Br···O interactions have previously been employed for crystal engineering purposes (Thallapally *et al.*, 2002). Halogen bonding between halogen atoms (Lewis acid) and neutral or anionic Lewis base, has been subject of great interest in recent years, primarily due to their unique noncovalent bonding characteristics (Metrangolo *et al.*, 2005; Riley *et al.*, 2009). The crystal structure additionally contains a C—H···π short contact, giving rise to an alternate linear pattern along the *a* axis (Table 1).

### Experimental

The title compound was prepared as follows: to a mixture of 1-(1,3-benzodioxol-5-yl)ethanone (1.64 g, 0.01 mol) and 2-bromobenzaldehyde (1.85 g, 0.01 mol) in 30 ml ethanol, 10 ml of 10 % sodium hydroxide solution was added and stirred at 5-10° C for 3 hours. The precipitate formed was collected by filtration and purified by recrystallization from ethanol. Final yield 79%; m.p. 390-392° K. Crystals suitable for X-ray analysis were grown from (1:1 v/v) mixture of toluene and acetone by slow evaporation method. Anal.: calc. for C_16_H_11_BrO_3_ : C 58.03 , H 3.35; found: C 57.93, H 3.31.

### Refinement

All H atoms were stereochemically fixed and refined using a riding option with C(Sp^2^)—H = 0.93 Å, C(methylene)—H = 0.97 Å, and U_iso_(H) = 1.2 U_eq_(C). The residual electron density observed in the vicinity of Br is due to the result of rotation of the bromophenyl moiety about the C6—C7 bond. The disorder could not be reliably refined presumably due to very low occupancy of other conformers. The crystal studied was treated as an inversion twin leading to twin fractions of 0.595 (13):0.405 (13).

### Crystal data

**Table:**

C_16_H_11_BrO_3_	*D*_x_ = 1.614 Mg m^−^^3^
*M**_r_* = 331.16	Melting point = 390–392 K
Orthorhombic, *P*2_1_2_1_2_1_	Mo *K*α radiation, λ = 0.71073 Å
Hall symbol: P 2ac 2ab	Cell parameters from 7488 reflections
*a* = 5.0434 (2) Å	θ = 2.5–25.7°
*b* = 12.9354 (4) Å	µ = 3.02 mm^−^^1^
*c* = 20.8916 (7) Å	*T* = 296 K
*V* = 1362.93 (8) Å^3^	Block, colorless
*Z* = 4	0.53 × 0.19 × 0.16 mm
*F*(000) = 664	

### Data collection

**Table:**

Bruker SMART APEXII CCD area-detector diffractometer	2674 independent reflections
Radiation source: fine-focus sealed tube	2437 reflections with *I* > 2σ(*I*)
graphite	*R*_int_ = 0.023
φ and ω scans	θ_max_ = 26.0°, θ_min_ = 1.9°
Absorption correction: multi-scan (*SADABS*; Bruker, 2004)	*h* = −6→6
*T*_min_ = 0.576, *T*_max_ = 0.653	*k* = −13→15
17031 measured reflections	*l* = −25→25

### Refinement

**Table:**

Refinement on *F*^2^	Secondary atom site location: difference Fourier map
Least-squares matrix: full	Hydrogen site location: inferred from neighbouring sites
*R*[*F*^2^ > 2σ(*F*^2^)] = 0.034	H-atom parameters constrained
*wR*(*F*^2^) = 0.086	*w* = 1/[σ^2^(*F*_o_^2^) + (0.0412*P*)^2^ + 0.7667*P*] where *P* = (*F*_o_^2^ + 2*F*_c_^2^)/3
*S* = 1.06	(Δ/σ)_max_ = 0.001
2674 reflections	Δρ_max_ = 0.64 e Å^−^^3^
182 parameters	Δρ_min_ = −0.51 e Å^−^^3^
0 restraints	Absolute structure: Flack (1983), 1086 Bijvoet pairs
Primary atom site location: structure-invariant direct methods	Flack parameter: 0.595 (13)

### Special details

**Table:**

Geometry. All esds (except the esd in the dihedral angle between two l.s. planes) are estimated using the full covariance matrix. The cell esds are taken into account individually in the estimation of esds in distances, angles and torsion angles; correlations between esds in cell parameters are only used when they are defined by crystal symmetry. An approximate (isotropic) treatment of cell esds is used for estimating esds involving l.s. planes.
Refinement. Refinement of *F*^2^ against ALL reflections. The weighted *R*-factor wR and goodness of fit *S* are based on *F*^2^, conventional *R*-factors *R* are based on *F*, with *F* set to zero for negative *F*^2^. The threshold expression of *F*^2^ > σ(*F*^2^) is used only for calculating *R*-factors(gt) etc. and is not relevant to the choice of reflections for refinement. *R*-factors based on *F*^2^ are statistically about twice as large as those based on *F*, and *R*- factors based on ALL data will be even larger.

### Fractional atomic coordinates and isotropic or equivalent isotropic displacement parameters (Å^2^)

**Table:**

	*x*	*y*	*z*	*U*_iso_*/*U*_eq_	
Br1	0.74414 (9)	0.52368 (3)	0.425085 (18)	0.07369 (16)	
O1	0.0651 (6)	0.5167 (2)	0.25271 (15)	0.0779 (9)	
O2	−0.6166 (5)	0.26394 (18)	0.06042 (12)	0.0586 (6)	
O3	−0.6165 (5)	0.44083 (18)	0.07678 (11)	0.0554 (5)	
C1	0.7744 (7)	0.3802 (2)	0.40661 (13)	0.0493 (7)	
C2	0.9616 (7)	0.3239 (3)	0.44092 (16)	0.0610 (9)	
H2	1.0660	0.3561	0.4717	0.073*	
C3	0.9903 (8)	0.2201 (3)	0.42877 (19)	0.0679 (11)	
H3	1.1121	0.1813	0.4520	0.081*	
C4	0.8394 (8)	0.1738 (3)	0.3824 (2)	0.0661 (10)	
H4	0.8595	0.1036	0.3741	0.079*	
C5	0.6594 (7)	0.2304 (3)	0.34812 (17)	0.0576 (9)	
H5	0.5603	0.1975	0.3166	0.069*	
C6	0.6196 (6)	0.3355 (3)	0.35892 (14)	0.0461 (7)	
C7	0.4282 (7)	0.3947 (3)	0.32148 (16)	0.0533 (8)	
H7	0.4276	0.4658	0.3280	0.064*	
C8	0.2596 (7)	0.3595 (2)	0.28037 (14)	0.0524 (7)	
H8	0.2561	0.2885	0.2732	0.063*	
C9	0.0717 (6)	0.4246 (3)	0.24404 (15)	0.0461 (7)	
C10	−0.1054 (6)	0.3754 (2)	0.19621 (13)	0.0413 (6)	
C11	−0.1108 (7)	0.2691 (3)	0.18603 (16)	0.0486 (7)	
H11	0.0015	0.2268	0.2097	0.058*	
C12	−0.2804 (7)	0.2245 (2)	0.14116 (15)	0.0522 (8)	
H12	−0.2842	0.1534	0.1347	0.063*	
C13	−0.4395 (6)	0.2889 (2)	0.10737 (14)	0.0436 (7)	
C14	−0.4375 (6)	0.3942 (2)	0.11713 (13)	0.0398 (6)	
C15	−0.2737 (7)	0.4401 (2)	0.16089 (12)	0.0430 (6)	
H15	−0.2739	0.5113	0.1670	0.052*	
C16	−0.7382 (8)	0.3593 (2)	0.04180 (13)	0.0498 (7)	
H16A	−0.7151	0.3704	−0.0038	0.060*	
H16B	−0.9267	0.3572	0.0510	0.060*	

### Atomic displacement parameters (Å^2^)

**Table:**

	*U*^11^	*U*^22^	*U*^33^	*U*^12^	*U*^13^	*U*^23^
Br1	0.0729 (2)	0.0700 (2)	0.0781 (3)	0.0085 (2)	−0.0259 (2)	−0.02381 (18)
O1	0.096 (2)	0.0487 (15)	0.0889 (19)	0.0021 (15)	−0.0448 (17)	−0.0083 (14)
O2	0.0621 (14)	0.0532 (13)	0.0605 (13)	0.0012 (11)	−0.0161 (12)	−0.0129 (11)
O3	0.0580 (13)	0.0492 (12)	0.0589 (13)	0.0062 (11)	−0.0187 (11)	−0.0013 (11)
C1	0.0454 (16)	0.0575 (17)	0.0449 (14)	−0.0019 (17)	0.0005 (14)	0.0032 (12)
C2	0.0490 (18)	0.088 (3)	0.0466 (17)	0.0060 (18)	−0.0039 (15)	0.0082 (18)
C3	0.057 (2)	0.083 (3)	0.064 (2)	0.0180 (19)	0.0026 (19)	0.027 (2)
C4	0.064 (2)	0.054 (2)	0.081 (2)	0.0069 (16)	0.0111 (19)	0.017 (2)
C5	0.056 (2)	0.056 (2)	0.0608 (19)	−0.0020 (15)	0.0003 (16)	0.0071 (17)
C6	0.0418 (15)	0.0552 (19)	0.0415 (15)	−0.0003 (15)	0.0029 (12)	0.0046 (13)
C7	0.0564 (19)	0.0461 (18)	0.0575 (18)	0.0046 (15)	−0.0111 (16)	−0.0041 (15)
C8	0.0509 (16)	0.0510 (16)	0.0553 (16)	−0.003 (2)	−0.0075 (18)	−0.0004 (13)
C9	0.0473 (17)	0.0479 (19)	0.0432 (15)	−0.0038 (15)	−0.0021 (13)	−0.0006 (13)
C10	0.0411 (15)	0.0450 (16)	0.0378 (13)	−0.0004 (13)	0.0037 (12)	0.0025 (13)
C11	0.0464 (17)	0.0473 (18)	0.0522 (17)	0.0055 (15)	−0.0052 (15)	0.0026 (15)
C12	0.058 (2)	0.0368 (14)	0.0621 (17)	−0.0016 (17)	−0.0073 (18)	−0.0041 (13)
C13	0.0435 (16)	0.0458 (17)	0.0415 (14)	−0.0033 (13)	0.0011 (13)	−0.0046 (13)
C14	0.0371 (14)	0.0441 (16)	0.0383 (13)	0.0011 (12)	0.0031 (12)	0.0026 (12)
C15	0.0463 (16)	0.0397 (13)	0.0429 (13)	−0.0003 (16)	−0.0004 (13)	−0.0022 (11)
C16	0.0492 (16)	0.0540 (17)	0.0463 (14)	−0.005 (2)	−0.0056 (16)	0.0007 (12)

### Geometric parameters (Å, °)

**Table:**

Br1—C1	1.902 (3)	C7—C8	1.292 (5)
O1—C9	1.206 (4)	C7—H7	0.9300
O2—C13	1.365 (4)	C8—C9	1.478 (5)
O2—C16	1.431 (4)	C8—H8	0.9300
O3—C14	1.374 (4)	C9—C10	1.484 (4)
O3—C16	1.423 (4)	C10—C11	1.391 (4)
C1—C2	1.391 (5)	C10—C15	1.402 (4)
C1—C6	1.392 (4)	C11—C12	1.394 (4)
C2—C3	1.374 (6)	C11—H11	0.9300
C2—H2	0.9300	C12—C13	1.355 (4)
C3—C4	1.371 (6)	C12—H12	0.9300
C3—H3	0.9300	C13—C14	1.378 (4)
C4—C5	1.368 (5)	C14—C15	1.368 (4)
C4—H4	0.9300	C15—H15	0.9300
C5—C6	1.393 (5)	C16—H16A	0.9700
C5—H5	0.9300	C16—H16B	0.9700
C6—C7	1.459 (4)		
			
C13—O2—C16	105.8 (2)	O1—C9—C10	120.6 (3)
C14—O3—C16	105.9 (2)	C8—C9—C10	119.1 (3)
C2—C1—C6	122.1 (3)	C11—C10—C15	119.9 (3)
C2—C1—Br1	117.4 (3)	C11—C10—C9	122.6 (3)
C6—C1—Br1	120.4 (2)	C15—C10—C9	117.5 (3)
C3—C2—C1	119.2 (4)	C10—C11—C12	121.6 (3)
C3—C2—H2	120.4	C10—C11—H11	119.2
C1—C2—H2	120.4	C12—C11—H11	119.2
C4—C3—C2	120.0 (3)	C13—C12—C11	117.3 (3)
C4—C3—H3	120.0	C13—C12—H12	121.3
C2—C3—H3	120.0	C11—C12—H12	121.3
C5—C4—C3	120.3 (4)	C12—C13—O2	128.1 (3)
C5—C4—H4	119.9	C12—C13—C14	121.8 (3)
C3—C4—H4	119.9	O2—C13—C14	110.2 (3)
C4—C5—C6	122.2 (4)	C15—C14—O3	128.0 (3)
C4—C5—H5	118.9	C15—C14—C13	122.2 (3)
C6—C5—H5	118.9	O3—C14—C13	109.8 (3)
C1—C6—C5	116.2 (3)	C14—C15—C10	117.3 (3)
C1—C6—C7	122.4 (3)	C14—C15—H15	121.4
C5—C6—C7	121.4 (3)	C10—C15—H15	121.4
C8—C7—C6	127.4 (3)	O3—C16—O2	108.3 (2)
C8—C7—H7	116.3	O3—C16—H16A	110.0
C6—C7—H7	116.3	O2—C16—H16A	110.0
C7—C8—C9	124.3 (3)	O3—C16—H16B	110.0
C7—C8—H8	117.9	O2—C16—H16B	110.0
C9—C8—H8	117.9	H16A—C16—H16B	108.4
O1—C9—C8	120.3 (3)		
			
C6—C1—C2—C3	−1.8 (5)	C15—C10—C11—C12	0.1 (5)
Br1—C1—C2—C3	−179.7 (3)	C9—C10—C11—C12	179.5 (3)
C1—C2—C3—C4	1.4 (5)	C10—C11—C12—C13	0.4 (5)
C2—C3—C4—C5	−0.2 (6)	C11—C12—C13—O2	178.6 (3)
C3—C4—C5—C6	−0.6 (6)	C11—C12—C13—C14	−0.7 (5)
C2—C1—C6—C5	1.0 (5)	C16—O2—C13—C12	178.4 (3)
Br1—C1—C6—C5	178.8 (2)	C16—O2—C13—C14	−2.3 (3)
C2—C1—C6—C7	−178.2 (3)	C16—O3—C14—C15	−179.3 (3)
Br1—C1—C6—C7	−0.4 (4)	C16—O3—C14—C13	1.5 (3)
C4—C5—C6—C1	0.2 (5)	C12—C13—C14—C15	0.6 (5)
C4—C5—C6—C7	179.4 (3)	O2—C13—C14—C15	−178.8 (3)
C1—C6—C7—C8	−173.5 (4)	C12—C13—C14—O3	180.0 (3)
C5—C6—C7—C8	7.4 (6)	O2—C13—C14—O3	0.5 (4)
C6—C7—C8—C9	−179.8 (3)	O3—C14—C15—C10	−179.3 (3)
C7—C8—C9—O1	−1.5 (6)	C13—C14—C15—C10	−0.1 (4)
C7—C8—C9—C10	177.6 (3)	C11—C10—C15—C14	−0.2 (4)
O1—C9—C10—C11	−177.9 (4)	C9—C10—C15—C14	−179.7 (3)
C8—C9—C10—C11	3.0 (4)	C14—O3—C16—O2	−2.8 (3)
O1—C9—C10—C15	1.5 (5)	C13—O2—C16—O3	3.1 (3)
C8—C9—C10—C15	−177.6 (3)		

### Hydrogen-bond geometry (Å, °)

**Table:**

Cg3 is the centroid of the C10–C15 ring.

**Table:**

*D*—H···*A*	*D*—H	H···*A*	*D*···*A*	*D*—H···*A*
C7—H7···Br1	0.93	2.69	3.164 (4)	113
C7—H7···O1	0.93	2.50	2.812 (6)	100
C16—H16B···Cg3^i^	0.97	2.76	3.563 (4)	141

Symmetry codes: (i) *x*−1, *y*, *z*.
